# Living in the Aftermath: Narratives on the impact of exposure to community and school violence in childhood on mental health and adjustment outcomes in later life

**DOI:** 10.1111/papt.70046

**Published:** 2026-02-16

**Authors:** Marinos Bomikazi Lupindo, Sam French, Paul Salkovskis

**Affiliations:** ^1^ Experimental Psychology Department University of Oxford Oxford UK; ^2^ Oxford Institute of Clinical Psychology Training University of Oxford, Warneford Hospital Oxford UK; ^3^ Oxford Health NHS Foundation Trust Oxford UK; ^4^ Warneford Hospital Oxford UK

**Keywords:** adjustment, childhood, community, mental health, school, South Africa, violence

## Abstract

**Objectives:**

To understand the impact of and subsequent reactions to exposure to extreme violence in young adults in South Africa exposed during school years. In particular, to get an in‐depth understanding of its immediate consequences and factors that ameliorate or exacerbate it.

**Design:**

An exploratory qualitative research design was used, using purposive sampling.

**Methods:**

Semi‐structured interviews with 21 young South African adults aged 19–31 were conducted online. Transcripts were analysed using thematic analysis.

**Results:**

Violence exposure was found to result in trauma reactions with themes of a continued sense of being unsafe, feeling damaged and defective because of having these reactions, and mistrust towards others. In terms of coping reactions, a theme of avoidance and/or reacting with aggressive behaviour was identified, which likely exacerbated the challenges they experienced. By contrast, a more positive theme was identified in some, focused on having a sense of community and connectedness, which was experienced as ameliorating these challenges.

**Conclusions:**

Exposure to community and school violence in childhood has a lasting impact on mental health and adjustment in later life. The impact is likely worsened by mistrust of others, a continued sense of feeling unsafe and counterproductive coping mechanisms, while connectedness and community seem to lessen the impact. Further research can refine these findings to build an understanding of these mechanisms to inform secondary prevention and treatment interventions in low‐ and middle‐income countries.

## INTRODUCTION

Exposure to adverse childhood experiences (ACEs) is a pervasive problem impacting childhood development, typically creating prolonged vulnerabilities that carry over into adulthood. According to McGrath et al. (2023) between 28.6% and 29.8% of individuals worldwide will experience mental illness in their lifetime, with the average onset of mental disorders being between the ages of 14 and 24 years. ACEs and social determinants (such as interaction with unsafe environments, peer and caregiver engagements, cultural and socioeconomic factors) have consistently been found to be associated with an early age of onset of mental health problems (Mngoma et al., 2021). Areas where extreme violence is more common, such as Palestine, Nigeria and South Africa, have been further identified as having the highest risk‐to‐prevalence ratios for mental disorders (Kessler et al., 2007).

Researchers attributed the early onset of mental health disorders to early life events that adversely impact the development of biological and psychosocial systems, as such circumstances can increase sensitivity to stress and impair psychological and physical well‐being (Hales et al., 2023; Nurius et al., 2015; Yıldız et al., 2023). Individuals exposed to a single experience of childhood adversity had increased risk of being exposed to multiple ACEs; such cumulative stressful life events further compromise psychosocial functioning (Lee et al., 2020; McGuigan & Middlemiss, 2005; Nurius et al., 2015; Storrie et al., 2023; Yıldız et al., 2023). It is therefore important to investigate context‐specific experiences of individuals in high‐risk countries such as South Africa to better understand the impact of ACEs in these regions, and how these progress into adulthood in order to inform prevention and intervention strategies. It is likely that historical factors and resultant distortion of infrastructure and social disruption will interact with broader psychological vulnerabilities.

The effect of factors such as colonialism more generally and apartheid (specifically in the case of South Africa) has been to adversely impact cultural and socioeconomic development. This is quite distinct from the situation in countries in the Global North, likely implying that consideration needs to be given to the generalizability and need for adaptation of mental health interventions developed across these different contexts and the need for specific research in those contexts. This is crucial as these histories have shaped the ongoing challenges and pre‐existing ways of coping with such adversities. It is clear that colonialism and apartheid in South Africa have resulted in continuing unequal access to education, racialised poverty and residential segregation. This is the outcome of the institutionalised racism that prioritised the hegemony and economic prosperity of White South Africans over Black, coloured^1^ (a distinct sociohistorical ethnic identity in South Africa, that is official and socially accepted as an ethnic descriptor, and includes a diverse ancestry including the Khoisan, Dutch, British and South‐East Asian and South Asian roots) and Indian South Africans (Levy, 2019, as cited in Abumere, 2024; Hoosen et al., 2022; Modise, 2025). The sequelae of these historical events continue to impact the socioeconomic landscape of South Africa as it is consistently reported as the world's unequal society with the highest Gini coefficient of 63% (Abumere, 2024) (representing the gap between highest and lowest income brackets in an economy). On a more positive note, there are African values that emphasise collective healing such as the concept of Ubuntu (humanity to others: I am what I am because of who we all are). Religion and spirituality are commonly deployed coping strategies in the face of adversity (Jansen et al., 2024); exploring and understanding these factors as potential protective factors in those exposed to violence may be crucial to conducting and interpreting research in this context.

Previous research has identified the impact that different forms of violence can have on subsequent life outcomes; such findings can inform interventions intended to mitigate adult vulnerabilities to adverse mental health outcomes (Al‐Modallal et al., 2020; Hales et al., 2023; Lee et al., 2020; McGuigan & Middlemiss, 2005; Nurius et al., 2015; Storrie et al., 2023; Vitek & Yeater, 2021; Yıldız et al., 2023). Childhood experiences of violence, abuse and neglect have been found to be associated with depression, anxiety disorders, substance use, suicidal behaviour and violence perpetration in later life (Lee et al., 2020; McGuigan & Middlemiss, 2005; Yıldız et al., 2023). However, these studies have typically focused on high‐income countries (HICs) (Miliauskas et al., 2022; Walker, 2021) with limited research focused on the lived experiences within low‐ and middle‐income countries (LMICs), where there are much higher levels of exposure to violence and other ACEs (Adewoye & du Plessis, 2021; Scorgie et al., 2017). Similarly, high levels of violence in LMICs such as South Africa are exacerbated by historical factors such as displacement in communities, with these displaced communities experiencing the highest levels of violence during the apartheid regime, continuing into the present (Modise, 2025). Such experiences are typically accepted and normalised (Scorgie et al., 2017). With the exception of Lee et al. (2020), such studies have focused on domestic violence and abuse, and family dysfunction rather than community violence which previous studies have identified as being the most common form of violence exposure in LMICs (Seedat et al., 2000, 2004). Research focused on understanding the lived experiences of individuals in LMICs experiencing violence and abuse represents an important gap, which if filled could provide insights into modifiable factors that moderate the impact of being exposed to ACEs.

There are a few studies which have used qualitative methods to gain an in‐depth understanding of individuals' experiences of violence exposure and the impact it has on them (Adewoye & du Plessis, 2021; García‐Montes et al., 2022; Scorgie et al., 2017; van Rosmalen‐Nooijens et al., 2017). They found significant and lasting physical, psychological and social impact that community and domestic violence had on survivors, including those who experienced violence in childhood and those whose violence experiences occurred later in life. Of note, both those directly victimised and those who witnessed violence expressed significant reluctance to seek help and share their experiences due to mistrust, fear of being re‐victimised and/or becoming the direct victims of the violence. Fears of being socially isolated due to being blamed for their violence experiences were also noted (Adewoye & du Plessis, 2021; García‐Montes et al., 2022; van Rosmalen‐Nooijens et al., 2017). Without adequate support, some individuals continue to struggle with the psychological effects of violence and experience isolation, while others become desensitised and/or become perpetrators of violence (Scorgie et al., 2017). Although these studies have provided some in‐depth insights, they did not elicit the psychological and social factors that lead to social isolation, mistrust of others, revictimisation and/or violence perpetration. Building knowledge in this area can provide insights into potential mechanisms that lead to poorer outcomes to inform targeted interventions for vulnerable individuals. The emphasis in the present study is to inform those promoting help‐seeking in contexts with high incidence of violence and resource constraints such as South Africa (Lund et al., 2009; World Health Organization (WHO), 2022).

The present interview study posed three research questions:

1. How is exposure to violence and its immediate consequences experienced by young people in South Africa?
2. How are different types of violence perceived as impacting later mental health and adjustment by individuals in the current study?
3. What factors are considered by those interviewed to ameliorate or exacerbate this impact?

## METHODS

### Design

The study used a qualitative design, conducting semi‐structured online interviews, in line with Levitt et al. (2018). We were interested in people's experiences of exposure to violence, its immediate consequences and how different types of violence are perceived as impacting later mental health and adjustment. Additionally, we wished to identify factors perceived as ameliorating and/or exacerbating this impact.

### Ethical considerations

Ethics approvals were received from the University of Oxford, in the United Kingdom, (CUREC: R86611/RE001) and Stellenbosch University, in South Africa, (HREC Reference No: N23/08/095) prior to the commencement of the study. Site access approval was also gained from the recruitment organisations. During ethics application and recruitment, additional considerations were made concerning legal disclosures. Particularly, participants were excluded from the study if their principal violence exposure was as perpetrators of violence and/or those who were incarcerated or in the process of being prosecuted.

Participant informed consent was obtained after providing full details of the study. Participants' identity and related data were kept anonymous. Data were securely stored in password protected files. Although the study did not anticipate putting participants at risk, debriefing was provided for all participants. The research protocol was overseen and evaluated by other South African‐based collaborating research psychologists. Clinical risk management (although it transpired that this was not required) was managed in line with the standards of the Health Professions Council of South Africa.

#### Description of participants and data collection site

Twenty‐one participants were recruited through Western Cape (South Africa)‐based mental health organisations. Participants were selected to include: (a) those who self‐identified as having experienced and were adversely affected by community and/or school violence that threatened or actually resulted in physical harm, (b) violence that occurred during the years they attended school and (c) they were currently aged 19–31 years of age (see Table 1 below for a summary of participants' description for final sample). The Western Cape was a suitable research site as it has been identified as having high rates of violence (Hiscox et al., 2021; Kaminer et al., 2013; Sherr et al., 2016).

**TABLE 1:** Summary of participants' description.

Characteristics	Frequency	Range/mean
Number of males	8	
Number of females	13	
Current participant age range	–	19–31 [*M* = 25.81 (*SD* = 3.98)]
Age rang at time of first violence experience	–	6–20 [*M* = 13.25 (*SD* = 3.10)]
Number of participants who identify as being Black	18	
Number of participants who identify as being Coloured^a^	3	
Common direct violence experiences		
Bullying	9	
Robbery and physical aggression with a weapon	8	
Sexual assault	6	
Common indirect violence experiences		
Aggressive forms of violence involving physical harm	9	
Mob justice^b^	4	
Commonly reported areas of residence when violence occurred		
Rural/urban area	0	
Townships^c^	21	

*Note*: See Appendix B Table B1 in Supporting Information for fully detailed description of participant characteristics.

^a^ April and Josias (2017, p. 31) describe the identity of ‘Coloured’ in South Africa as a diverse group, with ancestry that includes European settlers (mostly Dutch and British), slaves from South‐East Asia and East Africa, as well as the indigenous Khoisan people. By contrast, ‘Black’ typically was described as referring to ‘indigenous or native African’ populations.

^b^ Mob justice is violence perpetrated by community members against a suspected criminal and accounts for 5% of trauma hospital admissions in South Africa (Traynor et al., 2020).

^c^ Underdeveloped urban residential areas, primarily reserved for non‐whites (Africans, Coloureds and Indians) during Apartheid, located near or in ‘white‐only’ zones (National Treasury, 2007), which are characterised by low‐ to lower middle‐income families.

#### Procedures

Purposive sampling was used to recruit through channels that the organisations used to communicate with their clients, including in‐person, emails, posters and social media. The organisations were provided with participant recruitment material for various recruitment mediums. The same information was disseminated through posters and directly to organisations that had the ability to identify prospective participants who met criteria. Participants who, from the advertisements, self‐identified as being suitable for and interested in the study directly contacted the researcher through the details provided on the advertisements. Others submitted their names to the respective organisations and were then directly contacted by the researcher; all were then screened for eligibility. Interviews were conducted via secure video conferencing (Microsoft Teams). The interview schedule was developed collaboratively with South African adults who had a history of violence experiences explored in this study (see full interview schedule in Appendix A in Supporting Information). Pilot interviews (with a total of four adults) were conducted prior to the commencement of the study, which helped us develop and phrase questions that reflect care and sensitivity to very sensitive content and captured the nuanced experiences described. Consultations with three South African clinical psychologists, who currently work (as both clinicians and researchers) within the context with both children and adult clients with violence experiences, were consulted to provide feedback on the phrasing of the questions as well as explore whether the schedule captured participants' experiences sufficiently to address the research questions. Participants were given the opportunity to indicate their preferred language for the interview (between English, Afrikaans and isiXhosa). Each participant was interviewed for approximately one hour, and follow‐up interviews were conducted where necessary. In two instances where participants appeared to be distressed, the researcher, with the agreement of the participant, brought the interview to a close and provided appropriate support and follow‐up. In both instances, participants requested a second interview in which they were able to successfully engage with minimal distress.

A total of 45 individuals initially expressed interest in the study; however, due to non‐response (*n* = 18) or not meeting recruitment criteria (*n* = 6), 21 individuals participated in the interviews. Participant recruitment and data collection were stopped based on information power and when code and meaning saturation was reached (Braun & Clarke, 2019; Malterud et al., 2016). Prior to the interviews, participants received R20 airtime (equivalent to £1 credit loaded on the phone) ahead of the interview to buy data for the interview. Participants who completed participation were provided with a R250 (equivalent to £10) voucher as recompense.

#### Data analysis

Interviews from all participants were audio recorded and transcribed verbatim. All participant information was anonymised, and each were assigned participant numbers. The risk of participant identifiability was mitigated by obtaining consent for the use of selected demographic information and by limiting the level of personally specific detail reported. This included using broad categorical descriptors (e.g. professional, skilled trade worker, student or unemployed) rather than specific occupational titles, thereby maintaining participant anonymity.

The authors adopted a critical realist approach to the interview data, using inductive thematic analysis as outlined by Braun and Clarke (2006). This was on the assumption that the violence experienced by participants was rooted in reality but that the meanings were shaped by their sociocultural context. NVivo 14 qualitative analysis software was used to organise the transcribed data. Themes were identified, explored for connections and coded into themes and subthemes. Member checking was conducted on 14% of the transcripts by the second author to confirm credibility was conducted by the third author, and the reliability was within the substantial range (Cohen's Kappa = .77), suggesting that the second rater found the themes developed by the first author to be clear and distinct.

#### Reflexivity

The first author, who primarily conducted the research, reflected on her epistemological position as a Black South African woman, a practicing clinician, her professional history in research and positioning as a qualitative researcher throughout the study. Her familiarity with the South African context in terms of its history and current structural, social and economic challenges was reflected on, specifically the anticipated influence this may have on the data collection and analysis process. These potential biases were minimised by having reflective and second raters throughout the research process. The second and third authors were white British with limited experience of the South African context, but extensive discussions were carried out in order to familiarise them with this; the third author further visited South Africa, including visits to the Townships where the participants were from. On the contrary, having grown up in South Africa and having personal experiences of both South African communities and the school system allowed the first author to approach the data collection with a shared understanding and empathy that may have created a sense of safety for participants and allowed them to explore in‐depth aspects of these experiences based on an understanding of the context. In addition, the first author's ability to communicate fluently in at least four of the official languages may have helped facilitate building rapport with the participants (all interviews were conducted by the first author). Lastly, her experience as a clinician with experience in trauma‐focused therapeutic work in South Africa may have also helped to create a safe and contained space for the participants and further helped participants feel more trusting of the interview space. The remaining authors also reflected on their personal and epistemological positioning. The first rater had lived experiences within the South African context which we considered may have resulted in interpretive bias during the thematic analysis process. To ensure analytic rigour and fidelity, the second author, who did not have the same lived experiences, independently reviewed the data, coding and emerging themes to ensure the findings were grounded in participants' experiences. Further extensive discussions were had with all three authors to ensure that the analysis was in line with the critical realist orientation.

## RESULTS

### Descriptive theme: Characteristics of violence experiences

Participants described a wide range of violence experiences, with all (except three) having encountered multiple categories of violent events, either as direct victims, witnesses or both. Rates at which participants reported violence occurring within their communities were extremely high, but they regarded it as being ‘normal’. Given most participants had multiple exposures to different violence types, the specific violence type being referred to will be highlighted.

#### Direct experience of violence

Fourteen participants reported experiences of direct exposure to violence, ranging from threats of physical harm to actual physical harm. They included bullying (nine), robbery (eight) and sexual assault (six).

Participants described a range of bullying experiences, most of which occurred during secondary school and were mostly directed towards females. Bullying included physical threat/ harm, unwanted sexual gestures and emotional forms, and was perceived resulting from personal factors such as their socioeconomic status, physical appearance and peer‐related factors.

When I went to the bathrooms, they wrote about me on the walls… They would call me tiny and I'm not tiny. They didn't want to call me [Participant name] or my nickname [Participant nickname]… They will try to trip me sometimes. Sometimes they would take my books away and then I'll be looking for my books. So those in that was emotional abuse, emotional violence, because I was looking for my book and they'll make fun of me in class then everyone would be laughing at me [Participant 2, Female, 30, **direct bullying**]

Participants who reported robbery, incidents occurred with perpetrators using or threatening physical force; either through physical assault or having weapons used to threaten and/ or harm participants. Notably, all participants had more than one robbery occur, experienced these incidents at varying times of the day and framed it as universal, and it was inevitable that they would eventually be subjected to robbery due to the high incident levels.

So around here we can't carry a phone whether it's at night or during the day. We can't even walk alone, even during the day or at night. At some point I got my phone stolen. It was during the day round about this time I was going to go by bread and then someone just jumped me and took my phone. Later that same month I went to go buy bread again and that time I didn't have a phone, so I didn't have a phone to give to them, I got robbed again [by different people] but they did take the money that I have. [Participant 14, Female, 26, **direct robbery** and sexual assault; witnessed mob justice]

One participant reported being physically assaulted with a weapon by a community member that was unfamiliar to him; however, he was unaware of the intent behind the attack as he was not robbed.

I went to a party with a friend… So yeah, this guy said you he was looking for me and we didn't know what he wanted from me. So he came to us and approached me and wanted to fight me. And my friend got involved trying to stop the fight. Unfortunately, the guy managed to stab him in instead of me… and he died in that moment. [Participant 17, Male, 23; **direct physical assault**]

All participants who reported direct sexual assault were females, their perpetrators were known to them and described various forms of sexual assault ranging from unwanted sexual comments and gestures to penetrative rape. Notably, most participants had more than one encounter of sexual assault. Other participants acknowledged that some sexual acts they had were not consensual as they were often coerced or they had not consented to them.

I was 16, I was in grade 10 And this was a sexual assault by someone who stage in the neighbourhood. I met him at a party, like there was like a child in our neighbourhood who had a birthday party and we go there and unless we met and we exchanged numbers and. We like started talking and we like became friends or so I thought. And then this one time he invited me over to his place. So he was like, turned out, he was like older and in varsity And he invited me over to play and then he gave me something to drink and then things became blurry after that. And then when I came to, he was on top of me, basically like raping me. So like this was me like trying to get him off him, not getting off. He kept saying he's sorry. He's almost done. [Participant 7, Female, 29, direct bullying, robbery and **sexual assault**]

#### Indirect experience of violence

Twelve participants reported indirect experiences of violence exposure, which were characterised by excessive force and violence. These included mob justice (four), robbery (four), aggressive physical assaults (four) and sexual assault (one).

Indirect violence experiences witnessed by participants often posed high levels of threat or caused significant harm, sometimes even leading to death.

I knocked on the gate with the little R1 coin and she comes and I tell her I want airtime [calling credit for a mobile network] I give her the money and then she leaves to go fetch the airtime while she leaves to go fetch the airtime I start hearing screaming she screams and then gunshots and I see people running and jumping over the fence and the husband was still chasing them but I think he lost a lot of energy, a lot of blood, and he just collapsed right there on the floor. [Participant 16, Female, 22, **witnessed robbery**]

Some of these violent acts were perpetrated by gang members:

I was 16. So it was in the local tavern and random people just came in fired shots while I was still in the bathroom… I think 8 people died. I was still inside and I was the last one to come out when the police started coming in to investigate. [Participant 20, Male, 25, **witnessed gang related violence**]

Other violent acts were reported to be perpetrated by community members who were retaliating against the high levels of crime and violence:

When I was 15 I witnessed a mob justice incident in [township name]. A man had been accused of theft, and a crowd quickly gathered around him. So people started shouting and hitting him with sticks and stones. So I remember the man trying to shield himself with his hands, but the crowd was relentless. The violence escalated very quickly and it felt like everyone was caught up in a frenzy. [Participant 19, Male, 27, **witnessed mob justice**]

Although no males reported direct experiences of sexual assault, one participant reported joining a gang notorious for raping young males who become members of the gang. When further probed whether the experience happened to him, he expressed only witnessing these acts being perpetrated towards other members and he opted not to join.

I used to work with a 28 gang outside before I went to prison, I used to work with a 28, so I come to prison I became a 27… Ma'am that guys They even rape each other in prison, in prison of the 28, I saw for the first time because they wanted to make me a 28 by the time, but I the thing that I saw I said no, I don't want to be in this gang anymore. [Participant 10, Male, 26, **witnessed sexual assault** and robbery]

### Main themes

**FIGURE 1:**
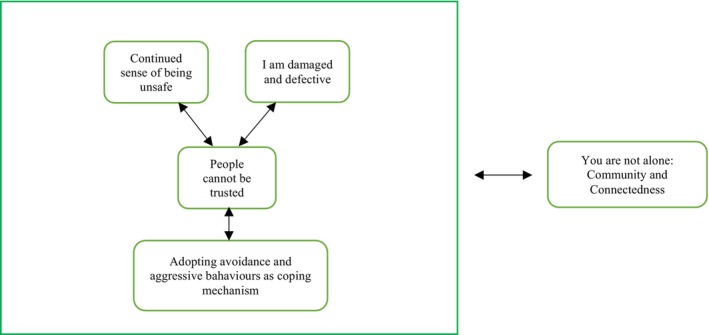
Summary of main themes.

Five main themes emerged from this study, and these themes have been summarised in Figure 1 below:

#### Impact of violence exposure

Below we describe themes around the impact that exposure to community and school violence had on participants within this study. The impact of these experiences spanned across various areas of functioning, including psychological, social and occupational areas, and in most instances progressed into adulthood. Strikingly, four participants [3, 8, 18 and 20] had not made clear associations between their experiences of violence in early life with their current adulthood challenges.

##### Continued sense of not being unsafe

Experiences of violence were reported to have a significant impact on participants resulting in psychological, social and occupational challenges. These challenges varied, including initial shock and fear of something more severe happening as an initial reaction. Notably, both direct and indirect forms of violence evoked similar emotions. For these individuals, the uncertainty of what might follow was paralysing. In the moment of exposure, they feared the situation would worsen, with the possibility of being physically harmed, killed or becoming a victim of the violence they were witnessing.

I guess I was scared. It was the first time that I was exposed to a gun and I perhaps I worried that I was going to die… but I remember for the second incident that you know, in that moment I was worried. I thought I was gonna die, I thought they were gonna rape us and I just froze. I didn't know what to do. [Participant 3, Female, 29, **direct robbery**; witnessed robbery]

Notably, participants reported that these initial trauma reactions persisted following the incidents of violence creating a continued sense of unsafety. Fifteen participants reported excessive anxiety presenting as paranoia, hypervigilance and fear that the incident might recur, while four participants experienced somatic symptoms including headaches, bowel‐related symptoms (e.g. cramps or diarrhoea) and nausea when exposed to triggers. For some (three), these symptoms were persistent and pervasive with only two participants [7 and 9] had their symptoms explained by a professional and started taking medication.

I was anxious to be in school (referring to bullying)… I had to start taking fluoxetine, cause I got to a point where I used to be scared to be by myself (referring to sexual assault). [Participant 9, Female, 26, **direct bullying**, robbery and **sexual assault**; witnessed mob justice]

Twelve participants reported that their trauma reactions, including flashbacks and anxiety, persisted into adulthood, continuing to impact their daily lives and decision making including deciding to drop out of school or job prospects. These intrusive memories affected their daily functioning, making them hypervigilant and causing distress, especially in scenarios reminiscent of their original trauma.

I'd feel safer when I was home. So some days I would just skip school and decide not to go. [Participant 18, Female, 26, direct bullying and **robber** **y**]

I hated going to the (taxi) rank so much because it brought back those flesh backs it was a reminder that happened out here, you know I didn't like sort of like a lot of males, it was all so difficult… I'm always anxious. [Participant 12, Female, 19, direct bullying and **sexual assault**]

Three participants [13, 15 and 18] further reported the significant impact that living in communities where there are high and continued incidents of violence exacerbates their fear of being unsafe.

I am a bit anxious if you can hear my voice. I am a bit anxious and I I stutter at the same time, so I I I think I still need to seek professional help because I I don't believe that I have healed from my previous traumas. I I do believe that even to date it still haunts me even though this happened over 10 years ago (referring to bullying experience)… Anything can happen, especially if you reside in such in such in neighbourhoods I fear for my life honestly speaking I do I I I fear for my life a lot (referring to robbery). [Participant 18, Female, 26, **direct bullying and robbery**]

Five participants described experiences of withdrawal and isolation following their violence exposure. They perceived this as stemming from profound low mood, along with a loss of interest and pleasure in life.

I would sleep a lot. So since now I've been reading a lot about depression, I can say I was also depressed. [Participant 2, Female, 30, **direct bullying**]

Only two participants [5 and 7] sought professional help, eventually being diagnosed with depression, though this help‐seeking behaviour occurred much later, during young adulthood. Notably, these experiences of depressed mood and subsequent diagnosis were exclusively reported by participants who struggled with self‐blame or felt singled out in their violence experiences. These depressive episodes, marked by withdrawal and isolation, were closely linked to participants' struggles with suicidal ideation and attempts.

By the time I was sitting in class I was not really consciously thinking about it and I found myself feeling sad and starting to cry [referring to bullying]… I remember I'd start crying that for the first time in my life I feel like a failure I carry the shame over my head. I carry the blame. I blamed myself a lot. Maybe I could have done better. I could have done things differently [referring to sexual assault]. [Participant 8, Female, 26, **direct bullying and sexual assault**]

##### I am damaged and defective

Six participants reported experiencing complete powerlessness and defeat during their trauma events which resulted from the awareness that they cannot overpower the perpetrator/s. This was also accompanied by thoughts and feelings of being weak, defective and/or accepting that bad things always happen to them, which confirmed (for 13 participants) pre‐existing beliefs from previous negative experiences [participants 2, 5, 6, 8 and 12] or developed as a result of the trauma and were further reinforced by events that followed.

I tried to fight him but I was like I'm just going to let him be. Like I accepted everything happening to me like I what's the point? You know, bad things always happen to you. I guess this is one of them. So I might as well just. I actually didn't feel anything. I was just numb like. [Participant 12, Female, 19, direct bullying and **sexual assault**]

I felt helpless, I felt paralysed and couldn't look away, despite actually wanting to… I wanted to actually intervene, but I realised that I don't think I have much power to actually intervene and and and help so hence that helplessness cut actually worked on my mind. [Participant 19, Male, 27, **witnessed mob justice**]

This brought feelings of shame causing them to isolate and conceal aspects of or the trauma in its entirety due to fear, anticipation and actual experiences of being ridiculed, blamed and/or reprimanded for their experiences.

If I tell them I'm I'm going to get into a lot of trouble because they're going to ask where I was going. Who's this guy that I was friends with, you know, they told me not to be friends with, with guys and to stay away from them so it was like that feeling that I'd be blamed as well on top of like me blaming myself. [Participant 7, Female, 26, direct bullying, robbery and **sexual assault**]

These beliefs were internalised and progressed into young adulthood resulting in negative self‐esteem, caused feelings of shame and left them in a continued state of powerlessness (10 participants).

Right now for me looking at it as rape and you know the concept rape, will I be able to to deal with myself? How will I look at myself with a be like girl you were raped!… But When now I have to do with someone else just as in I I just don't have the energy, I just don't have the solution for that for all of it. [Participant 8, Female, 27, direct bullying and **sexual assault**]

Notably, three participants [5, 8 and 9] attributed their vulnerability to trauma as a result of the initial isolation and perceived rejection from others following past adversities as keeping them in a cycle of violence through revictimisation (as victims and perpetrators). Additionally, in the instance of seven participants, anxiety and fear affected their employability and/or their ability to retain employment as a result of their trauma‐related challenges.

So I had a problem with the attachment, I got so attached because even after, he did start some things that I felt that I was not comfortable with this but I always looked at me and I thought maybe it's me that's doing this thing wrong because I looked at the fact that I never had a father and I never had a mother and I kept on making these mistakes. [Participant 5, Female, 26, **direct sexual assault**]

##### People cannot be trusted

Seven participants reported feeling shock, anger and/or disbelief towards their perpetrators as they created a false sense of safety but later betrayed them. These betrayals ranged from being bullied or sexually assaulted by friends to being betrayed by seemingly concerned harmless strangers.

But the second time it's more of the betrayal thing that you're talking about that the first time it happened, it was someone that I trusted but for the second time I was more disappointed than angry. So I was really disappointed not even angry. [Participant 14, Female, 26, **direct sexual assault** and robbery; witnessed mob justice]

Nine participants felt the betrayal significantly affected their ability to trust others even in adulthood. They concealed their trauma due to difficulty trusting people with their feelings and experiences in fear that these would be used against them and increase risk to further harm to themselves or others. Notably, in instances of gang‐related violence including robbery, seven participants reported this was further reinforced by others discouraging participants from sharing their experiences with them in fear that they will be implicated and therefore personally targeted by violence perpetrators.

No, ma'am, I can't just talk to someone that I don't know. I must talk with someone I can trust with my personal information. I can't just talk to anyone… Yes, I keep it to myself because I can't open, like, talk to that guy. Now I tell that guy tomorrow that guy can tell or which one come to tell that one oh that guy is like that. No, I can't just talk to anyone and I just keep it for myself. [Participant 10, Male, 26, **witnessed robbery**]

#### Factors exacerbating the impact of violence exposure

Below we describe strategies participants adopted to manage their violence‐related challenges. These include avoidance strategies used as an attempt to distract from their experiences, numbing (through substance use) or completely detaching from trauma‐related emotions and individuals, which increased risk to cyclic experiences of violence towards self and/or others, sometimes reinforcing feelings of guilt and shame. However, these were reported to be ineffective as participants always found themselves having the same feelings and triggers when these strategies were not used.

##### Adopting avoidance and aggressive behaviours as a coping mechanism

Eight participants described deliberately isolating themselves, avoiding social interactions and staying away from places or people that could trigger their anxiety as a way to feel safer.

I started to isolate myself. I was actually, I loved playing in the streets, you know, with my fellow friends playing soccer. But after the incident, I was always at home, you know, watching TV, I didn't really want to go outside. I only go outside if I go to school or actually go to the shop. So I isolated myself purposefully because of the incident. I was very scared of the streets. [Participant 1, Male, 23, **witnessed mob justice**]

Eighteen participants spoke about emotionally detaching from their experiences due to how chronic (frequency and severity) violence occurs within these contexts or finding ways to distract from or dismiss their feelings through substance use, sleeping, social media or extracurriculars.

I kind of my brain kinda just suppressed it the same way it suppressed the first time it happened, but I was not thinking about it most of the time… I'm a workaholic so it helped me because I was I was doing so well and participating in so many sports extracurricular activities, my mind was always occupied, so although I would think about it, it wouldn't be for a long time period. So I was either doing something or I was sleeping [Participant 14, Female, 26, direct robbery and **sexual assault**; witnessed mob justice]

Participants also described how the embodied detachment was also a protective mechanism as exhibiting fear further increased risk of being victimised.

That experience, it has shaped me in a way, into trying to survive because when you live in this area, fear does not help, showing them that you are scared doesn't help. You have to put up an act somehow to survive because even a small kid would take advantage of you if they see your reaction. [Participant 15, female, 29, **witnessed robbery**]

Notably, participants who witnessed mob justice had conflicting feelings including having empathy for the victim but also relief and detachment as the victim allegedly terrorised the community. These conflicting feelings were observed to occur amongst participants with multiple violence experiences prior to witnessing mob justice. By contrast, Participant one who reported mob justice as his first exposure to violence reported significant psychological impact.

In the morning I had to get water with a bucket and he was literally lying there, people are jumping his body and what can I do? I fetched the water and I was just. I looked at him, he was lying dead. Dead! What can I do? I get my water and I go boil the water. I bathed and I went to campus. [Participant 9, Female, 26, direct bullying, robbery and sexual assault; **witnessed mob justice**]

Similarly, eight participants described internalising violence, becoming verbally aggressive towards others and/or becoming physical perpetrators of violence in attempt to protect themselves. Four participants joined gangs as an attempt to seek protection from them and avoid future harm. However, this resulted in substance use and escalation of violence perpetration that participants found difficult to leave as they were threatened with death being the only way out, further perpetuating a cycle of violence and fear.

I would be very rude because I wanted people to fear and, what is this word, when you come to me, you know your story! You know that you cannot just do anything to me because you know, I'm that girl! So I stopped being a soft girl. [Participant 8, Female, 27, **direct bullying** and sexual assault]

Participant 10 and 11 reported this violence escalation further having significant dire consequences as they dropped out and/ or got expelled from school and eventually became imprisoned further reinforcing feelings of shame and guilt.

I was in grade 6 and I would bully the children also, they would bully me as well. I felt like I must bully you… In grade 7 I could do everything at school, I hit the children almost everyday. I want to beat the teacher, I want to beat anyone at that time.… I beat the children in school and the teachers said to me [participant name] you can't do that. And I do it again every time, you see, and they expelled me from the school and it was very tough though. [Participant 11, Male, 23, **direct bullying**]

#### Factors that ameliorate the impact of violence

Below we describe experiences participants found to be helpful for them to deal with and/or recover from their traumas. These included experiences of connectedness that re‐instilled a sense of psychological safety. Notably, the theme highlights experiences participants *felt* would be helpful, with very few participants having had actual experiences of community and connectedness.

##### Community and connectedness: You are not alone

Twenty participants described supportive environments (such as school, peers, family or professional mental health support) that were accepting of them following their trauma would have made a significant difference.

More support maybe acknowledging that you experience this, not just Oh, it happens, don't talk about it because but maybe give it is to speak more about it. So that I'm supported and I'm safe, not only when I'm home, when I feel like, OK, maybe I'm safe here, but going to school, being at school being supported at this and we are assuring you that you are safe, it's gonna it's just those those ways to say, listen, we've got you, you are safe. We are here now. Nothing's gonna happen. That reassurance, I think, would have helped. But it never happened. [Participant 15, female, 29, **witnessed robbery**]

Participants who shared their experiences and were met with compassion and support found that having their feelings validated and their experiences reframed helped them build confidence and resilience.

Talking to them (referring to friends) made me feel better cause I could sleep at night without overthinking, and I didn't have to cry myself to sleep. And I I could go out again… but definitely it made me feel like I'm not really the one who's in the wrong. [Participant 12, Female, 19, direct bullying and **sexual assault**]

But just because I can talk about it, it doesn't mean that it never makes me sad. It still makes me sad, but I've learned it's it's part of my story and I still don't understand why why it happened to me but in a crazy, twisted way, it shaped the brave person that I am. [Participant 9, Female, 26, direct bullying, robbery and **sexual assault**; witnessed mob justice]

Six participants added that having others who have went through similar adversities allowed them to make sense of, and feel validated in, their emotional reactions, thus, helping them better process their trauma. Participants who were able to access mental health support programmes reported gaining an understanding of expected trauma reactions and learned adaptive coping skills improving their ability to cope (with their own experiences and further helped others cope) and seek support for past and future adversities.

After the incident, you know, I I spoke to people because I believe speaking to people actually helps, you know, lessen the pain initially, I didn't talk to anyone because I didn't know how to process what I had seen, however I mentioned it to my best friend, who also lived in [township name] and had witnessed similar events… another significant experience was volunteering with a youth mentorship programme, you know, helping kids, you know, younger kids navigate their own challenges, you know, gave me a sense of purpose and helped me focus on positive contributions. [Participant 19, Male, 27, **witnessed mob justice**]

They further added that finding a home in art, music and creative writing allowed them to create a safe outlet for their experiences.

I thought let me just have a journal and then tried to deal with these things in a different way and maybe after having the journal then I will start… I realise I need to put my thoughts down, write everything that I go through reflect on it. Eventually I want to have something where I can share those things, now we can start speaking about them and even write a book. [Participant 5, Female, 26, **direct sexual assault**]

## DISCUSSION

This study sought to investigate the range of experiences related to exposure to community and school violence in young people in South Africa and the immediate consequences of this exposure. The most common direct victimisation experiences reported in this sample were bullying, robbery, physical aggression with a weapon and sexual assault, while indirect experiences (witnessing) were mainly aggressive forms of violence involving physical harm, including robbery, physical assault and mob justice. Identified themes related to the immediate psychological, social and occupational impact of violence and its long‐lasting effects. These themes included struggling with a continued sense of feeling unsafe as a result of ongoing trauma reactions, feeling damaged and defective, being mistrustful towards others and maladaptive coping strategies, including avoidance and internalising violence. Participants were also able to reflect on experiences that made and/or would have made the impact of their experiences better. These included feeling that they are not alone in their experiences and having renewing and repairing their sense of safety (physical and emotional/psychological).

There was evidence that different types of violence/abuse experiences varied in their impact. The *common features* were that violence exposure typically results in trauma reactions, and the meaning made from both the experiences and the resulting impact likely influenced the progression of challenges into adulthood. The main *differences* were that individuals who experienced violence where they felt singled out (specifically in cases of bullying and sexual assault) tended to experience significant shame, mood‐related symptoms and were more likely to conceal their experiences. By contrast, ‘shared’ traumatic events such as robbery were normalised and participants were able to better cope with the impact of these experiences, albeit males tended to experience feelings of shame when they struggled with lasting trauma reactions and related challenges. We were further able to identify several factors which were perceived as having either beneficial or adverse impacts on the consequences of violence exposure. Beneficial factors were social support from others, the awareness that others have been through similar experiences and related challenges, and unconditional positive regard from the significant people in their network. This resulted in feelings of being socially accepted, improving the long‐term impact of violence exposure. By contrast, negative meaning making about the self, others and the world, and actual or perceived experiences of rejection and isolation following trauma worsened the symptoms and outcomes.

The current study's findings are consistent with previous research on the impact of exposure to community and school violence. Additionally, there were several aspects that contributed to knowledge in this area which were consistent with or extended previous findings. Firstly, consistent with many studies investigating the topic, the study found that violence exposure has significant and lasting impact across various areas of functioning (Centre for Disease Control (CDC), 2024; Norman et al., 2012; Stansfeld et al., 2017). This included increased risk of trauma, mood and anxiety disorders such as depression and PTSD. However, individuals in low‐ and middle‐income countries exposed to violence in childhood have previously been found to have low access to support services (Lund et al., 2009; World Health Organization (WHO), 2022), increasing their risk of poor life outcomes (Dhungana et al., 2022; Downey & Crummy, 2022) consistent with findings in the present study. Notably, in relation to previous research indicating that males may present with less depression and PTSD symptoms and milder severity compared with females (Alisic et al., 2014; Hiscox et al., 2021; Nothling et al., 2017; Stansfeld et al., 2017), males in this study were more likely than females to share about indirect forms of violence and concealed the significant impact their direct experiences had due to shame for struggling following such adversities. Recent studies conducted in the United States of America have also begun highlighting the role males' tendency to conceal mental health challenges may have on the current the understanding and landscape of gender‐related differences observed in mental health problems (Isaksson et al., 2024; Smith et al., 2018). In this study, concealment and/or detachment from the psychological impact of trauma was reported to be a protective mechanism from future violence victimisation for both males and females, specifically in high and continued violence contexts. This further negatively impacted participants' outcomes as they internalised negative perceptions about themselves, others and the world thus maintaining their trauma reactions, consistent with current cognitive models of PTSD (Ehlers & Clark, 2000). In addition, the study's findings that cognitive processes during the trauma (such as feelings of powerlessness and confusion) contribute to the maintenance of trauma reactions was consistent with Dunmore et al.'s (2001) findings. Dunmore et al. (2001) aimed to expand on Ehlers and Clark's (2000) cognitive model of PTSD by highlighting the role of mental defeat, detachment and mental confusion, during physical and sexual assault, in maintaining PTSD. This highlights consistencies in factors that contribute to the development and maintenance of PTSD in samples found in the Global North and South. It may be, however, that there are differences in how this sense of safety is re‐established, as highlighted below.

Secondly, violence exposure has previously been found to have a significant impact on occupational outcomes. This may be a consequence of reduced interest in school, challenges concentrating due to trauma‐related stress which affects academic performance, as well as truancy (Adewoye & du Plessis, 2021; Foa et al., 2018). Participants in the current study reported similar outcomes; additionally, as an attempt to seek protection from victimisation, they either became violence perpetrators themselves and/or were involved with perpetrators resulting in challenging behaviours that lead to academic exclusion through being expelled or dropping out. Lastly, although previous research has highlighted the moderating effects of social support, resilience and positive school climate on the impact of violence exposure (Miliauskas et al., 2022; Walker, 2021), these studies did not provide an indication of the processes that result in these outcomes. In a nuanced finding that contributes to this understanding from the present study is that violence was perpetrated by people participants trusted and felt safe with thus adding an additional element of *betrayal* to the consequences of the violent act. This resulted in anticipation that people cannot be trusted, leading to withdrawal and isolation or violence perpetration as self‐protective measures which exacerbated trauma‐related challenges and worsened outcomes. The development of distrust can thus result in them distancing themselves from those who might otherwise offer support. By contrast, individuals who were able to re‐establish this sense of trust and safety through connectedness and acceptance with others who provided validation and reframing their experiences appeared to have more resilience in the face of past, present and felt equipped for future aversities.

There are several strengths and limitations to this study. The study investigated multiple forms of violence exposure, their related impact, as well as factors that make this better or worse. This is an area which has a growing body of research but very limited insights into the impact of different forms of violence and psychological processes that mediate and/or moderate violence exposure. The qualitative approach allowed for in‐depth insights into an area and setting that have limited previous research on psychological processes that can inform interventions. Participants were offered interviews in a language they would express themselves best, albeit that the majority opted to be interviewed in English.

The sample was a convenience sample recruited through mental health organisations within the Western Cape and was predominantly female, which could mean the themes were not fully representative of males. However, the sample was purposive in that it consisted of individuals from various regions and socioeconomic statuses and may be moderately representative of the Western Cape. Although the study was limited by its retrospective stance, participants were asked to reflect on experiences which tend to stick in their minds and reported being able to reflect on both past and present experiences of violence and abuse. A second rater coded 14% of the transcripts to gauge the reliability of the thematic coding, and analytic rigour may have been improved through double coding a larger proportion of the transcripts. However, the interrater reliability for the second‐rated transcripts was high, suggesting that the coding may be reliable. Lastly, the majority of participants in this study reported not engaging in any help‐seeking process, which may have limited the study's ability to assess barriers to help seeking.

There are several clinical implications that can be drawn from the findings of the current study. Firstly, it was evident that many of the participants who were exposed to violence continued to struggle in adulthood, which may suggest the need for both secondary and tertiary interventions for youth and young adults living in high violence contexts. Very few participants had any access to evidence‐based trauma‐focused mental health support services, suggesting an urgent need to increase support and accessibility to support services to school‐going children and young adults. It may also be beneficial to deliver these interventions in and/or through school‐based programmes facilitated by schools and/or other organisations working with school‐going aged children. Participants' expression of the dire consequence of not understanding common trauma reactions further suggests a need for psychoeducation aimed at improving mental health literacy, reducing stigma and advocating for support of individuals exposed to violence.

Future research would benefit from evaluating the impact of exposure to violence at the time of occurrence and the use of longitudinal designs. The progression of the challenges experienced into young adulthood while individuals have little to no support suggests an urgent need to understand the barriers to accessing professional support. In addition, although participants in the study highlighted barriers to gaining social support from their peers and their families, it may be beneficial to build knowledge on how these support structures can be strengthened without leaving both participants and their supporters feeling burdened by the knowledge of participants' violence experiences. It may also be beneficial to further develop and investigate benefits of targeted school‐based support and early interventions which participants in this study highlighted as having the potential to improve their outcomes. However, as previously noted, there is very little research into the effectiveness of these interventions, and the value of targeting mediating and moderating factors. Therefore, there is a need for extending the current qualitative research through quantitative research to further investigate the psychological processes involved in the mediation and moderation of mental health and adjustment outcomes following violence exposure. Such research will further inform secondary prevention interventions for at‐risk adolescents as well as informing tertiary interventions for young adults who were exposed to violence in early life and present with persisting challenges.

## CONCLUSION

The current study adds to the current body of knowledge on the impact of community and school violence in childhood on mental health and adjustment in later life by adding in‐depth and nuanced experiences shared by participants. These insights help build understanding of the mechanisms involved as they relate to factors that make the impact of violence exposure better or worse. Through early identification and intervention, the life outcomes of young people living in high and continued violence context may be improved (du Plessis et al., 2015; Hlungwani, 2015; Schwartz et al., 2021).

## AUTHOR CONTRIBUTIONS

**Marinos Bomikazi Lupindo:** Conceptualization; investigation; writing – original draft; methodology; writing – review and editing; software; formal analysis; project administration; data curation; visualization. **Sam French:** Validation; writing – review and editing; software. **Paul Salkovskis:** Conceptualization; validation; methodology; writing – review and editing; supervision.

## CONFLICT OF INTEREST STATEMENT

The research has no conflict of interests.

## Supporting information


Data S1.

## Data Availability

Due to the sensitive nature of the current research, data will not be made available to protect information that can be used to identify participants in this study.

## Footnotes

^1^
April and Josias (2017, p. 31) describe the identity of ‘Coloured’ in South Africa as a diverse group, with ancestry that includes European settlers (mostly Dutch and British), slaves from South‐East Asia and East Africa, as well as the indigenous Khoisan people. By contrast, ‘Black’ typically was described as referring to ‘indigenous or native African’ populations.

## References

[papt70046-bib-0001] Abumere, F. A. (2024). Colonial and apartheid legacy: Social, economic, and political inequality in South Africa. In Monuments and memory in Africa (1st ed., pp. 127–143). Routledge. 10.4324/9781003432876-8

[papt70046-bib-0002] Adewoye, S. E. , & du Plessis, A. (2021). Early adolescent bystanders' experiences of school bullying in a South African school. Journal of Education, 85, 111–127. 10.17159/2520-9868/i85a06

[papt70046-bib-0003] Alisic, E. , Zalta, A. K. , van Wesel, F. , Larsen, S. E. , Hafstad, G. S. , Hassanpour, K. , & Smid, G. E. (2014). Rates of post‐traumatic stress disorder in trauma‐exposed children and adolescents: Meta‐analysis. British Journal of Psychiatry, 204, 335–340. 10.1192/bjp.bp.113.131227 24785767

[papt70046-bib-0004] Al‐Modallal, H. , Al‐Omari, H. , Hamaideh, S. , & Shehab, T. (2020). Childhood domestic violence as an ancestor for adulthood mental health problems: Experiences of Jordanian women. The Family Journal, 28(4), 390–395. 10.1177/1066480720909845

[papt70046-bib-0005] April, K. A. , & Josias, A. (2017). Diasporic double consciousness – Creolized identity of colored professionals in South Africa. Effective Executive, 20(4), 31–61.

[papt70046-bib-0006] Braun, V. , & Clarke, V. (2006). Using thematic analysis in psychology. Qualitative Research in Psychology, 3(2), 77–101. 10.1191/1478088706qp063oa

[papt70046-bib-0007] Braun, V. , & Clarke, V. (2019). To saturate or not to saturate? Questioning data saturation as a useful concept for thematic analysis and sample‐size rationales. Qualitative Research in Sport, Exercise and Health, 13(2), 201–216. 10.1080/2159676X.2019.1704846

[papt70046-bib-0008] Centre for Disease Control (CDC) . (2024, July 19). Preventing Youth Violence |Violence Prevention|Injury Center|CDC . https://www.cdc.gov/violenceprevention/youthviolence/fastfact.html

[papt70046-bib-0009] Dhungana, S. , Koirala, R. , Ojha, S. P. , & Thapa, S. B. (2022). Association of childhood trauma, and resilience, with quality of life in patients seeking treatment at a psychiatry outpatient: A cross‐sectional study from Nepal. PLoS One, 17(10), e0275637. 10.1371/journal.pone.0275637 36194614 PMC9531790

[papt70046-bib-0010] Downey, C. , & Crummy, A. (2022). The impact of childhood trauma on children's wellbeing and adult behavior. European Journal of Trauma & Dissociation, 6(1), 100237. 10.1016/j.ejtd.2021.100237

[papt70046-bib-0011] du Plessis, B. , Kaminer, D. , Hardy, A. , & Benjamin, A. (2015). The contribution of different forms of violence exposure to internalizing and externalizing symptoms among young South African adolescents. Child Abuse & Neglect, 45, 80–89. 10.1016/j.chiabu.2015.02.021 25804436

[papt70046-bib-0012] Dunmore, E. , Clark, D. M. , & Ehlers, A. (2001). A prospective investigation of the role of cognitive factors in persistent Posttraumatic Stress Disorder (PTSD) after physical or sexual assault. Behaviour Research and Therapy, 39(9), 1063–1084. 10.1016/S0005-7967(00)00088-7 11520012

[papt70046-bib-0013] Ehlers, A. , & Clark, D. M. (2000). A cognitive model of posttraumatic stress disorder. Behaviour Research and Therapy, 38, 319–345. 10.1016/s0005-7967(99)00123-0 10761279

[papt70046-bib-0014] Foa, E. B. , Asnaani, A. , Zang, Y. , Capaldi, S. , & Yeh, R. (2018). Psychometrics of the Child PTSD Symptom Scale for DSM‐5 for Trauma‐Exposed Children and Adolescents. Journal of Clinical Child and Adolescent Psychology, 47(1), 38–46. 10.1080/15374416.2017.1350962 28820616

[papt70046-bib-0015] García‐Montes, R. , Fares‐Medina, S. , Diaz‐Caro, I. , Corral‐Liria, I. , & García‐Gómez‐Heras, S. (2022). The impact of violence on women's health. The present as a reflection of the past: A qualitative study. PLoS One, 17(9), e0273973. 10.1371/journal.pone.0273973 36084074 PMC9462808

[papt70046-bib-0016] Hales, G. K. , Saribaz, Z. E. , Debowska, A. , & Rowe, R. (2023). Links of adversity in childhood with mental and physical health outcomes: A systematic review of longitudinal mediating and moderating mechanisms. Trauma, Violence & Abuse, 24(3), 1465–1482. 10.1177/15248380221075087 PMC10240645 35226575

[papt70046-bib-0017] Hiscox, L. V. , Hiller, R. , Fraser, A. , Rabie, S. , Stewart, J. , Seedat, S. , Tomlinson, M. , & Halligan, S. L. (2021). Sex differences in post‐traumatic stress disorder in a high adversity cohort of south African adolescents: An examination of depressive symptoms, age, and trauma type as explanatory factors. European Journal of Psychotraumatology, 12(1), 1978669. 10.1080/20008198.2021.1978669 34691370 PMC8530480

[papt70046-bib-0018] Hlungwani, T. M. (2015). Prevalence and Predictors of Psychosocial Outcomes Amongst Socioeconomically Deprived Primary School Children in a Rural Setting in South Africa: The Role of Ecological Factors . [Ph.D., University of the Witwatersrand, Johannesburg (South Africa)]. https://www.proquest.com/docview/3157242465/abstract/DC2F0803ADAA46E6PQ/1

[papt70046-bib-0019] Hoosen, P. , Adams, S. , Tiliouine, H. , & Savahl, S. (2022). Youth and adolescents' perceptions of violence in post‐apartheid South Africa: A systematic review of the literature. Child Indicators Research, 15(3), 885–911. 10.1007/s12187-021-09890-5 35069928 PMC8767533

[papt70046-bib-0020] Isaksson, J. , Nyman, S. , Schwab‐Stone, M. , Stickley, A. , & Ruchkin, V. (2024). The severity of perceived stress associated with community violence exposure and its role in future posttraumatic stress: Findings from a longitudinal study of U.S. adolescents. Child and Adolescent Psychiatry and Mental Health, 18(1), 121. 10.1186/s13034-024-00813-0 39322966 PMC11423508

[papt70046-bib-0021] Jansen, S. , Niyonzima, J. B. , Gerbarg, P. , Brown, R. P. , Nsengiyumva, A. , Niyonsenga, J. , & Nsabimana, E. (2024). Evaluating effects of community‐based social healing model on Ubuntu, mental health and psychosocial functioning in post‐genocide Rwanda: Protocol for cluster randomized control trial. Current Controlled Trials in Cardiovascular Medicine, 25(1), 773. 10.1186/s13063-024-08632-6 PMC11568552 39550586

[papt70046-bib-0022] Kaminer, D. , Hardy, A. , Heath, K. , Mosdell, J. , & Bawa, U. (2013). Gender patterns in the contribution of different types of violence to posttraumatic stress symptoms among South African urban youth. Child Abuse & Neglect, 37(5), 320–330. 10.1016/j.chiabu.2012.12.011 23357516

[papt70046-bib-0023] Kessler, R. C. , Angermeyer, M. , Anthony, J. C. , De Graaf, R. , Demyttenaere, K. , Gasquet, I. , De Girolamo, G. , Gluzman, S. , Gureje, O. , Haro, J. M. , Kawakami, N. , Karam, A. , Levinson, D. , Medina‐Mora, M. E. , Oakley‐Browne, M. A. , Posada‐Villa, J. , Stein, D. J. , Adley Tsang, C. H. , Aguilar‐Gaxiola, S. , & Üstün, T. B. (2007). Lifetime prevalence and age‐of‐onset distributions of mental disorders in the World Health Organization's World Mental Health Survey Initiative. World Psychiatry, 6(3), 168–176. 18188442 PMC2174588

[papt70046-bib-0024] Lee, H. , Kim, Y. , & Terry, J. (2020). Adverse childhood experiences (ACEs) on mental disorders in young adulthood: Latent classes and community violence exposure. Preventive Medicine, 134, 106039. 10.1016/j.ypmed.2020.106039 32097756

[papt70046-bib-0025] Levitt, H. M. , Bamberg, M. , Creswell, J. W. , Frost, D. M. , Josselson, R. , & Suárez‐Orozco, C. (2018). Journal article reporting standards for qualitative primary, qualitative meta‐analytic, and mixed methods research in psychology: The APA Publications and Communications Board task force report . https://discovery.ucl.ac.uk/id/eprint/10042196 10.1037/amp0000151 29345485

[papt70046-bib-0026] Lund, C. , Boyce, G. , Flisher, A. J. , Kafaar, Z. , & Dawes, A. (2009). Scaling up child and adolescent mental health services in South Africa: Human resource requirements and costs. Journal of Child Psychology and Psychiatry, 50(9), 1121–1130. 10.1111/j.1469-7610.2009.02078.x 19243477

[papt70046-bib-0027] Malterud, K. , Siersma, V. D. , & Guassora, A. D. (2016). Sample size in qualitative interview studies: Guided by information power. Qualitative Health Research, 26(13), 1753–1760. 10.1177/1049732315617444 26613970

[papt70046-bib-0028] McGrath, J. J. , Al‐Hamzawi, A. , Alonso, J. , Altwaijri, Y. , Andrade, L. H. , Bromet, E. J. , Bruffaerts, R. , de Almeida, J. M. C. , Chardoul, S. , Chiu, W. T. , Degenhardt, L. , Demler, O. V. , Ferry, F. , Gureje, O. , Haro, J. M. , Karam, E. G. , Karam, G. , Khaled, S. M. , Kovess‐Masfety, V. , … Zaslavsky, A. M. (2023). Age of onset and cumulative risk of mental disorders: A cross‐national analysis of population surveys from 29 countries. The Lancet Psychiatry, 10(9), 668–681. 10.1016/S2215-0366(23)00193-1 37531964 PMC10529120

[papt70046-bib-0029] McGuigan, W. M. , & Middlemiss, W. (2005). Sexual abuse in childhood and interpersonal violence in adulthood: A cumulative impact on depressive symptoms in women. Journal of Interpersonal Violence, 20(10), 1271–1287. 10.1177/0886260505278107 16162489

[papt70046-bib-0030] Miliauskas, C. R. , Faus, D. P. , da Cruz, V. L. , do Nascimento Vallaperde, J. G. R. , Junger, W. , & Lopes, C. S. (2022). Community violence and internalizing mental health symptoms in adolescents: A systematic review. BMC Psychiatry, 22(1), 253. 10.1186/s12888-022-03873-8 35397541 PMC8994919

[papt70046-bib-0031] Mngoma, N. F. , Ayonrinde, O. A. , Fergus, S. , Jeeves, A. H. , & Jolly, R. J. (2021). Distress, desperation and despair: Anxiety, depression and suicidality among rural south African youth. International Review of Psychiatry, 33(1–2), 64–74. 10.1080/09540261.2020.1741846 32310008

[papt70046-bib-0032] Modise, J. M. (2025). The legacy of apartheid: Lasting inequalities and their contribution to crime. Multinational Research Society Journal, 2, 28–37.

[papt70046-bib-0033] National Treasury . (2007). Townships in the South African geographic landscape . Department of Provincial and Local Government.

[papt70046-bib-0034] Norman, R. E. , Byambaa, M. , De, R. , Butchart, A. , Scott, J. , & Vos, T. (2012). The long‐term health consequences of child physical abuse, emotional abuse, and neglect: A systematic review and meta‐analysis. PLoS Medicine, 9(11), e1001349. 10.1371/journal.pmed.1001349 23209385 PMC3507962

[papt70046-bib-0035] Nothling, J. , Simmons, C. , Suliman, S. , & Seedat, S. (2017). Trauma type as a conditional risk factor for posttraumatic stress disorder in a referred clinic sample of adolescents. Comprehensive Psychiatry, 76, 138–146. 10.1016/j.comppsych.2017.05.001 28521252

[papt70046-bib-0036] Nurius, P. S. , Green, S. , Logan‐Greene, P. , & Borja, S. (2015). Life course pathways of adverse childhood experiences toward adult psychological well‐being: A stress process analysis. Child Abuse & Neglect, 45, 143–153. 10.1016/j.chiabu.2015.03.008 25846195 PMC4470711

[papt70046-bib-0037] Schwartz, B. , Kaminer, D. , Hardy, A. , Nothling, J. , & Seedat, S. (2021). Gender differences in the violence exposure types that predict PTSD and depression in adolescents. Journal of Interpersonal Violence, 36(17–18), 8358–8381. 10.1177/0886260519849691 31130044

[papt70046-bib-0038] Scorgie, F. , Baron, D. , Stadler, J. , Venables, E. , Brahmbhatt, H. , Mmari, K. , & Delany‐Moretlwe, S. (2017). From fear to resilience: Adolescents' experiences of violence in inner‐city Johannesburg, South Africa (special issue: Urban health at the edge: A series on reproductive health and HIV in inner‐city Johannesburg.). BMC Public Health, 17, 51–64. 10.1186/s12889-017-4349-x 28832282 PMC5498857

[papt70046-bib-0039] Seedat, S. , Nyamai, C. , Njenga, F. , Vythilingum, B. , & Stein, D. J. (2004). Trauma exposure and post‐traumatic stress symptoms in urban African schools. Survey in CapeTown and Nairobi. The British Journal of Psychiatry, 1, 169–175. 10.1192/bjp.184.2.169 14754831

[papt70046-bib-0040] Seedat, S. , van Nood, E. , Vythilingum, B. , Stein, D. J. , & Kaminer, D. (2000). School survey of exposure to violence and posttraumatic stress symptoms in adolescents. Southern African Journal of Child and Adolescent Mental Health, 12(1), 38–44. 10.1080/16826108.2000.9632366

[papt70046-bib-0041] Sherr, L. , Hensels, I. S. , Skeen, S. , Tomlinson, M. , Roberts, K. J. , & Macedo, A. (2016). Exposure to violence predicts poor educational outcomes in young children in South Africa and Malawi. International Health, 8(1), 36–43. 10.1093/inthealth/ihv070 26678567 PMC4716801

[papt70046-bib-0042] Smith, D. T. , Mouzon, D. M. , & Elliott, M. (2018). Reviewing the assumptions about Men's mental health: An exploration of the gender binary. American Journal of Men's Health, 12(1), 78–89. 10.1177/1557988316630953 PMC5734543 26864440

[papt70046-bib-0043] Stansfeld, S. A. , Rothon, C. , Das‐Munshi, J. , Mathews, C. , Adams, A. , Clark, C. , & Lund, C. (2017). Exposure to violence and mental health of adolescents: South African health and well‐being study. BJPsych Open, 3(5), 257–264. 10.1192/bjpo.bp.117.004861 29093828 PMC5643877

[papt70046-bib-0044] Storrie, C. L. , Kitissou, K. , & Messina, A. (2023). The effects of severe childhood physical and sexual abuse on adult socioeconomic prosperity. Journal of Child & Adolescent Trauma, 16(1), 55–68. 10.1007/s40653-022-00499-6 36776634 PMC9908797

[papt70046-bib-0045] Traynor, M. D. , Laing, G. L. , Bruce, J. L. , Hernandez, M. C. , Kong, V. Y. , Rivera, M. , Zielinski, M. D. , & Clarke, D. L. (2020). Mob justice in South Africa: A comparison of blunt trauma secondary to community and non‐community assaults. Injury, 51(8), 1791–1797. 10.1016/j.injury.2020.04.014 32475650

[papt70046-bib-0046] van Rosmalen‐Nooijens, K. A. , Lo Fo Wong, S. H. , Prins, J. B. , & Lagro‐Janssen, A. L. (2017). The need for control, safety and trust in healthcare: A qualitative study among adolescents and young adults exposed to family violence. Elsevier Ireland Ltd. 10.1016/j.pec.2017.02.008 28238419

[papt70046-bib-0047] Vitek, K. N. , & Yeater, E. A. (2021). The association between a history of sexual violence and romantic relationship functioning: A systematic review. Trauma, Violence & Abuse, 22(5), 1221–1232. 10.1177/1524838020915615 32242504

[papt70046-bib-0048] Walker, D. (2021). Vicarious victimization, negative emotions, and maladaptive coping: Investigating the role of violent peers. Violence and Victims, 36(1), 45–65. 10.1891/VV-D-19-00142 33328341 PMC12903200

[papt70046-bib-0049] World Health Organization (WHO) . (2022, October 12). Barriers to mental health care in Africa . WHO | Regional Office for Africa. https://www.afro.who.int/news/barriers‐mental‐health‐care‐africa

[papt70046-bib-0050] Yıldız, M. , Orak, U. , & Aydoğdu, R. (2023). Enduring effects of early life traumas on adult suicidal ideation. Journal of Child & Adolescent Trauma, 16(2), 297–307. 10.1007/s40653-022-00482-1 37234841 PMC10205958

