# Supplementary material for: Living in the Aftermath: Narratives on the impact of exposure to community and school violence in childhood on mental health and adjustment outcomes in later life
Source: Psychol Psychother. 2026 Feb 16;99(2):600–20. doi: 10.1111/papt.70046 (PMC13162185; doi:10.1111/papt.70046)
Supplement: Supplementary file 1 — Data S1. [file PAPT-99-600-s001.zip › papt70046-sup-0002-Supinfo2@Supplementary material 24_11_2025.docx]

| University of Oxford, Department of Experimental Psychology  Anna Watts Building, Woodstock Road, Oxford, OX2 6GG  Tel: +44(0)1865271444  Main Supervisor: Paul Salkovskis  [paul.salkovskis@hmc.ox.ac.uk](mailto:paul.salkovskis@hmc.ox.ac.uk)  Principal Investigator: Marinos Bomikazi Lupindo  marinos.lupindo@sjc.ox.ac.uk |  | 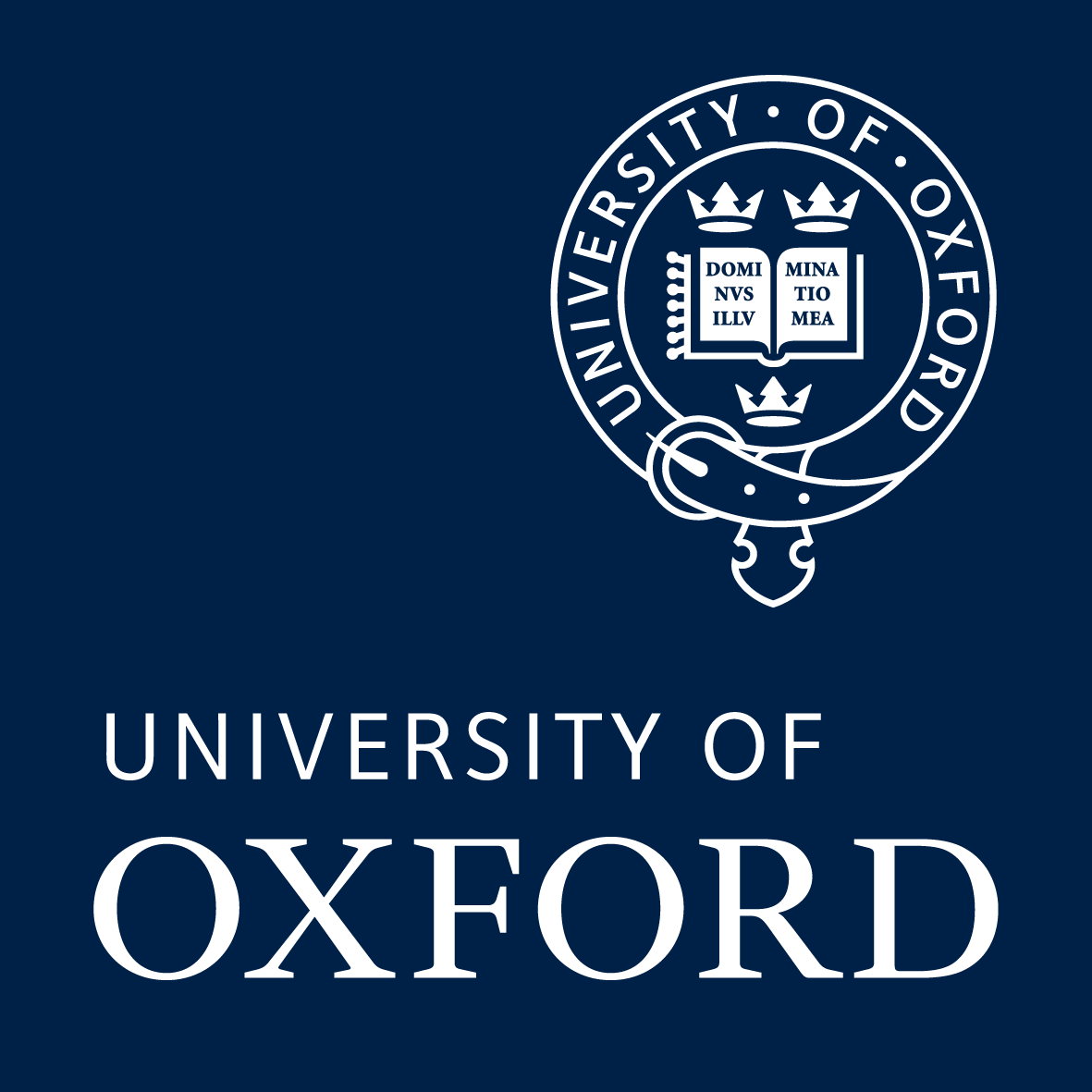 |
| --- | --- | --- |

**APPENDIX A: INTERVIEW SCHEDULE**

**UNIVERSITY OF OXFORD**

Central University Research Ethics Committee (CUREC) Approval Reference: R86611/RE001

University of Stellenbosch (HREC Reference No: N23/08/095)

**Impact of exposure to community/ school violence during school going years on mental health and adjustment in later life**

**Interview schedule**

Give brief description of the study and what the purpose of the interview is. Reiterate confidentiality and that the identity of the participant will be protected.

Five minutes at the beginning of the interview will be spent on rapport building with participants.

When asking questions, distinguish between experiences with family, friends, and acquaintances.

1. Tell me a bit about yourself. How old are you, what do you do and what are your current living circumstances? How do you spend your time? (Probe to locate person in personal and professional environment)

Ndixalele kancinci ngawe. Uneminyaka emingaphi? Wenzani ukuziphilisa? Isimo sakho sempilo sime kanjani? Ixesha lakho ulichita ngokwenzani?

Vertel vir my n bietjie meer van jouself. Hoe oud is jy, wat doen jy op die huidige oomblik, wat is jou huidige lewens omstandigede? Wat is jou belangstellings, hobbies en roetine? Waaraan spandeer jy die meeste tyd?

1. We are particularly interested in people’s reactions to violent and abusive experiences that may have happened during school years and how that might have affected you. Is it okay if I ask you more about this?

Sifuna ukwazi kabanzi ngodlame nokuhlukumezeka okungenzeka lwehlekele abantu ngesxhesha lokufunda isikolo nanokuthi kubaphatha kanjani abantu futhi kubaphazamisa kanjani. Ngabe ayikho inxaki xa ndiqhubeka ngokukubuza ngalonto?

Ons studie is geinteresseerd in mense se reaksie oor ervarings oor geveldadigheid en mishandeling, wat tydens skoolyare mag gebeur het, en hoe dit jou in die verlede en nou geaffekteer het. Is dit reg as ek jou meer hieroor vra?

1. Can you tell me about experiences that happened earlier in your schooling life when you might have witnessed or been involved in violent or abusive experiences? (Probes as necessary: we are looking for what happened, reactions at the time and reactions afterwards)

Wake wacaphazeleka kudlame nokuhlukumezeka okujongane nawe ngexesha usafunda isikolo? Ngicela undicazele kabanzi ngalento.

Kan jy my meer vertel van ervarings wat vroeier in jou skoollewe gebeur het, toe jy ervarings van geweld en mishandeling gesien het, of by betrokke by was?

1. At the time when this/ these episode/s were happening, what was your reaction (emotional and behavioural)? What did you think would happen to you?

Ngelixesha kwenzeka lento wawuziva kanjani? Yini owawuyenza ukujongana nalemekho? Yini eyayifika enqondweni wakho? Wawucinga ukuthi kuzokwenzakalani kuwe ngeloxesha?

Op die tydstip toe hierdie episode/episodes plaas gevind het, wat was jou reaksie (emosinele en gedrag)? Wat dink jy sou met jou gebeur het?

1. After this had happened, how were you? How did it make you feel? Can you describe your thoughts/feelings and behaviours?

Emva kwaleso sehlakalo, ungasichaza kanjani isimo owawukuso? Ingabe waziva kanjani? Ungazichaza incingo zakho, nemizwa kanye nendlela owawenza ngayo?

Na jou ervaring, hoe het dit vir jou laat voel? Kan jy jou gedagtes/gevoelens en gedrag beskryf?

1. As you retell the story, what’s happening for you right now, what are you feeling? Are you getting the same reactions you had at the time and does it feel like the incident is replaying in your mind?

Njengoba uphinda uyibalisa lendaba, kwenzakalani kuwena ngoku? Uziva kanjani? Ngabe uzifumana uziva sengathi ukulesimo ngokwemizwa owawukuso ngexesha kwenzeka lesosigameko? Ingabe isithombe sesigameko siyabuya yini enqondweni?

Terwyl jy die storie oorvertel, wat gebeur nou vir jou, hoe voel jy? Kry jy dieselfde reaksie wat jy destyds gehad het? En voel dit as of die voorval in jou gedagtes herhaal?

1. At the time of the experience, how did/ do you cope with the thoughts and feelings that you were experiencing? How are you coping now?

Ngexesha kwenzeka lesosgameko kuwe, wakwazi kanjani ukumelana nencinga nemizwa owawunayo ngaloxesha? Ngabe uqhuba kanjani ngoku?

Ten tye van die ervaring, hoe het jy die gedagtes en gevoelens wat ervaar het hanteer? Hoe hanteer jy dit nou?

1. Did you share this experience with anyone at the time? Did you share your experiences after the incident? Why/ why not?

Ngabe ukhona umuntu owabalisela ngalesigameko owadlula kuso? Ngabe ukhona owamxoxela ngendlela owaphazamiseka ngayo ngemuva kwalesi sigameko? Kungani?

Het jy hierdie ervaring/ervarings destyds, onmiddelike na die veoorval met iemand gedeel? Hoe lank na die voorval het jy dit met iemand gedeel? Hoekom het jy/hoekom het jy nie?

1. When you shared this, how did they react to it?

Ngesikhathi ubaxoxela ngalento, bayithatha kanjani?

Hoe het hulle daarop gereakgeer toe jy die met hulle deel?

1. In terms of your own and other people’s reactions, when you look back, were there things that were helpful/ unhelpful?

Xa ujonga emuva indlela owathatha ngayo isimo nangendlela abantu abasithatha ngayo, ngabe zikhona izinto ezaziluncedo nezazingancedisani nesimo sakho?

Waneer jy terug kyk, in terme van jou eie, en ander mense se reaksies, was behulpsame of nuttige bydrae/insette?

1. Are there things that you now think would have been helpful at the time? (Probe: own and others’ reactions)

Ngabe zikhona izinto ocinga ukuthi zazingaba luncedo ngalexesha?

Is daar dinge wat jy nou dink wat op daardie stadium behelpsaam/nuttig sou wees?

1. Is there anything else you would like to share with me that would help me better understand your experience?

Ngabe kukhona into ongathanda ukungixalela yona engandinceda ukuthi ndiqonde kancono isimo sakho?

Is daar enige iets anders wat jy met my wil deel om jou ervarings beter te verstaan?

1. Is there anything that you would like to ask me?

Ingabe kukhona ongathanda ukungibuza kona?

Is daar dalk enige iets wat jy vir my wou vra?

1. How are you feeling now? (If participant feels worse than at the start if the interview, provide debriefing and potentially offer a follow up session with them)

Uziva kanjani ngoku?

Hoe voel jy nou?
