# Supplementary material for: Living in the Aftermath: Narratives on the impact of exposure to community and school violence in childhood on mental health and adjustment outcomes in later life
Source: Psychol Psychother. 2026 Feb 16;99(2):600–20. doi: 10.1111/papt.70046 (PMC13162185; doi:10.1111/papt.70046)
Supplement: Supplementary file 1 — Data S1. [file PAPT-99-600-s001.zip › Clean Participant demographics.docx]

**APPENDIX B**

**Participants Demographics Table B1**

| **Participant code** | **Gender** | **Age** | **Ethnicity** | **Highest level of education** | **Occupation** | **Relationship status** | **Age of first violence experience** | **Exposed to single or multiple violences** | **Received psychological services of MH** | **Received Medication for MH** | **Violence exposure type** |
| --- | --- | --- | --- | --- | --- | --- | --- | --- | --- | --- | --- |
| Participant 1 | Male | 23 | Black | Diploma | Professional | Single | 6 or 7 years old | Multiple | No | No | Mob justice |
| Participant 2 | Female | 30 | Black | Grade 12 | Unemployed | Single | 14 years old | Multiple | No | No | Bullying and robbery |
| Participant 3 | Female | 29 | Black | Masters | Professional | In a relationship | 9 years old | Multiple | Yes | No | Robbery direct and witnessed |
| Participant 4 | Male | 29 | Coloured | Grade 12 | Skilled trade worker | In a relationship | 10 or 11 years old | Multiple | No | No | Physical assault witness |
| Participant 5 | Female | 26 | Black | Grade 12 | Undergraduate student | Single | 16 years old | Multiple | Yes | Yes | Bullying, sexual assault |
| Participant 6 | Female | 26 | Black | Masters | Professional | Single |  | Single | Yes | No | Bullying |
| Participant 7 | Female | 29 | Black | Postgraduate Diploma | Professional | Single | 9 years old | Multiple | Yes | Yes | Robbery, sexual assault, bullying |
| Participant 8 | Female | 27 | Black | Postgraduate degree | Unemployed | In a relationship | 14 years old | Multiple |  |  | Bullying, sexual assault |
| Participant 9 | Female | 26 | Black | Masters | Professional | In a relationship | 15/16 years old | Multiple | Yes | No | Robbery, sexual assault, bullying, witnessed mob justice |
| Participant 10 | Male | 26 | Coloured | Grade 9 | Unemployed | Single | 13 years old | Multiple | No | No | Witnessed robbery |
| Participant 11 | Male | 23 | Coloured | Grade 7 | Skilled trade worker | Single | 12 years old | Multiple |  |  | Bullying |
| Participant 12 | Female | 19 | Black | Grade 12 | Unemployed | In a relationship | 7 years old | Multiple | No | No | Bullying, sexual assault |
| Participant 13 | Male | 27 | Black | Masters | Professional | In a relationship | 17 years old | Multiple | Yes | No | Robbery |
| Participant 14 | Female | 26 | Black | Grade 12 | Undergraduate student and skilled trade worker | Single | 13 years old | Multiple | Yes | Yes | Sexual assault, robbery, mob justice |
| Participant 15 | Female | 29 | Black | Further Education and Training Certificate | Professional | Single | 15/16 years old | Multiple | No | No | Witnessed robbery |
| Participant 16 | Female | 22 | Black | Undergraduate degree | Undergraduate student | In a relationship | 17 years old | Multiple | Yes | No | Witnessed robbery |
| Participant 17 | Male | 23 | Black | Grade 12 | Unemployed | Single | 20 years old | Multiple | Yes | No | Physical aggression |
| Participant 18 | Female | 26 | Black | Grade 12 | Undergraduate student and skilled trade worker | Single | 13 years old | Multiple | No | No | Bullying, robbery |
| Participant 19 | Male | 27 | Black | Masters | Professional | In a relationship | 15 years | Multiple | Yes | No | Witnessed mob justice |
| Participant 20 | Male | 25 | Black | Grade 12 | Unemployed | In a relationship | 16 years old | Multiple | No | No | Witnessed gang related violence |
| Participant 21 | Female | 20 | Black | Grade 12 | Unemployed | Single | 10 years | Multiple (same type) | No | No | Direct bullying and witnessed physical assault |
